# Editorial: Protozoa and Their Hosts: An Oxidative Relationship

**DOI:** 10.3389/fcimb.2022.856191

**Published:** 2022-02-08

**Authors:** Rubem Menna-Barreto, Lucia Piacenza, Nisha J. Garg

**Affiliations:** ^1^ Laboratório de Biologia Celular, Instituto Oswaldo Cruz, Fundação Oswaldo Cruz, Rio de Janeiro, Brazil; ^2^ Departamento de Bioquímica and Centro de Investigaciones Biomédicas (CEINBIO), Facultad de Medicina, Universidad de la República, Montevideo, Uruguay; ^3^ Department of Microbiology & Immunology, University of Texas Medical Branch, Galveston, TX, United States

**Keywords:** protozoa, invertebrate host, vertebrate host, protozoa-host cell interactions, oxidative stress

The balance between the protozoans virulence and host immune responses (invertebrates and/or vertebrates) is crucial for the successful completion of the life cycle of these parasites in their hosts. Strategies such as macromolecules secretion, resistance to oxidative species, and silencing of macrophages, among many others, are applied by the parasites to evade host defenses. In this context, redox balance and biochemical mechanisms implicated by the parasitic protozoans represent a promising starting point for the development of novel anti-parasitic drugs and vaccines, and for testing their efficacy in preclinical and clinical trials. Reactive oxygen species (ROS) participate in regulating the protozoans’ infection asis extensively reported in the literature. Antioxidant enzymes expressed by the parasites play a biological role as virulence factors, contributing to the complexity of the parasite-host relationship. In this Research Topic, cellular, molecular, and biochemical aspects of the interactions of pathogenic protozoans and their hosts with a focus on parasite survival, dissemination, and evasion of host immune responses, and the suggestion of novel strategies for parasite elimination were addressed.

This Research Topic comprises six articles, three of which were original contributions. Velásquez et al. from Justus Liebig Universität Gießen (Giessen, Germany) presented the concept of macromeront formation by the apicomplexan protozoa *Eimeria bovis*, a parasite of veterinary importance, in the primary host endothelial cells. The authors showed that *E. bovis* promoted an important alteration in the glycolytic metabolism and mitochondrial plasticity of the host cells. Increased glucose and pyruvate consumption associated with high lactate production indicated a pivotal role of glycolysis in infected cells, data confirmed by the use of glycolytic inhibitors. Increased ROS generation and altered membrane potential suggests mitochondrial dysfunction in macromeront-carrying host cells. In another contribution developed by the researchers at the Universidad de la República (Montevideo, Uruguay), Specker et al. showed the role of mitochondrial peroxiredoxin (MPX) of the parasite *Trypanosoma cruzi*, the causative agent of Chagas disease, in determining the infectivity in macrophages and attenuating the toxicity of the anti-*T. cruzi* drug nifurtimox. Parasites with enhanced content of MPX were more resistant to nifurtimox and this was not due to the efficient peroxidase activity but instead a new holdase activity of this peroxiredoxin. MPX holdase activity was characterized in this study, and shown to increase in parasites stressed with drug challenge (nifurtimox and benznidazole) and temperature (37°C). The authors’ findings point to a parasite response to protein unfolding and degradation, highlighting the importance of maintaining parasite proteastasis. The relative contribution of peroxidase *versus* holdase activity in macrophage infection was evaluated using an inhibitor of the peroxidase but not holdase activity (ConoidinA). Increase in parasite MPX content enhanced infection whereas Conoidin A treated parasites had lower infection index, demonstrating the crucial role of MPX peroxidase activity to overcome the reactive oxygen and nitrogen species generated in the macrophage phagosome.

Studying the immunity of the mosquito *Aedes aegypti* during the infection with the trypanosomatid *Strigomonas culicis*, Bombaça et al. from Instituto Oswaldo Cruz (FIOCRUZ, Rio de Janeiro, Brazil) demonstrated that the presence of this protozoan led to an alteration in redox metabolism of the *A. aegypti* midgut, compromising insect reproductive fitness. As main findings, the H_2_O_2_-resistant strain of the trypanosomatid induced high levels of peroxidesproduction by dual oxidase activity that impaired the antioxidant system, while wild-type isolate of *S. culicis* signaled hydrogen peroxide release *via* mitochondrial metabolism of midgut cells. Both strains of the trypanosomatid impacted the reproductive fitness by the disruption of fecundity and fertility of the mosquitoes, providing novel biological aspects of *A. aegypti*-*S. culicis* interactions that can be used for the development of alternative vector control strategies.

In relation to the three review articles presented in this Research Topic, Vasquez et al. from New York University (Grossman School of Medicine, New York, United States) reviewed the role of oxidative stress during pathogenesis of malaria, caused by *Plasmodium* species. The upregulation of host oxidative enzymes such as xanthine oxidase as well as parasitic degradation of hemoglobin, among other aspects, and their contribution to the severity of malarial complications were discussed. The maintenance of oxidative balance in malaria patients was suggested as a therapeutic approach in preventing the lethal manifestation of this disease. Libisch et al. from Institut Pasteur de Montevideo (Montevideo, Uruguay) reviewed the literature on transcriptional changes during *T. cruzi* infection of host cells. Authors noted that the complexity and diversity of transcriptomic changes depends on the parasite virulence and cell types. Yet a few key points were identified, and authors discussed in detail the changes in host respiratory chain and oxidative phosphorylation in response to infection, concluding that the host responses act independently of the parasite strain, cell type, and experimental conditions. Lastly, Wan and Garg from University of Texas Medical Branch (Galveston, Texas, United States) reviewed the mitochondrial dysfunction, oxidative stress and inflammation in Chagas disease and discussed the role of sirtuins in mitigating these pathologies. Authors noted that reactive oxygen and nitrogen species produced by proinflammatory reactions in acute phase are essential to directly kill the pathogen. Yet, mitochondrial damage, a major cause of high ROS levels in the myocardium during chronic phase of the disease, was deemed as a major determinant of Chagas disease pathology. These authors also discussed the potential of sirtuin 1 agonists in improving the mitochondrial function and arresting oxidative and inflammatory stress in *T. cruzi* infection.

## Author Contributions

RM-B drafted the first version. RM-B, LP, and NG wrote the manuscript. All authors approved and reviewed the manuscript.

## Conflict of Interest

The authors declare that the research was conducted in the absence of any commercial or financial relationships that could be construed as a potential conflict of interest.

## Publisher’s Note

All claims expressed in this article are solely those of the authors and do not necessarily represent those of their affiliated organizations, or those of the publisher, the editors and the reviewers. Any product that may be evaluated in this article, or claim that may be made by its manufacturer, is not guaranteed or endorsed by the publisher.

